# Investigating angiotensin II concentration in modulating *Plasmodium falciparum* infection and angiotensin receptor activation

**DOI:** 10.3389/fpara.2026.1852476

**Published:** 2026-08-10

**Authors:** Auley De, Aparna Tiwari, Medha Agarwal, Veena Pande, Abhinav Sinha

**Affiliations:** 1 Department of Epidemiology, Indian Council of Medical Research (ICMR)-National Institute of Malaria Research, New Delhi, India; 2 Department of Biotechnology, Bhimtal, Kumaun University, Uttarakhand, India; 3 Department of Epidemiology, Academy of Scientific and Innovative Research, Ghaziabad, UP, India

**Keywords:** angiotensin II, AT1 and AT2 receptors, malaria, *Plasmodium falciparum*, receptor activation

## Abstract

**Introduction:**

Angiotensin II (Ang II) has been proposed to protect from severe malaria by killing the parasite and maintaining the blood-brain barrier integrity by acting through type 1 (AT_1_R) and type 2 (AT_2_R) receptors. However, this protection from severe malaria is counterproductive in adulthood as the elevated plasma Ang II puts the individual at risk for developing hypertension. Various natural and synthetic peptides of Ang II have been explored to check its antiplasmodial actions despite Ang II being a significant vasopressor via its actions through AT_1_R. Therefore, to be able to explore Ang II as a potential antiplasmodial agent, it is important to demonstrate its IC_50_ against *P. falciparum in vitro* and its minimum effective concentration required for activation of AT_1_R. It is desirable that IC_50_ of Ang II against *P. falciparum* remains below the threshold for AT_1_R activation and hence to verify this, the current study was planned as there are no previously published reports for determining the threshold Ang II concentration for AT_1_R activation.

**Methods:**

For invasion inhibition assay, Pf-infected RBCs (schizonts) were incubated with four different concentrations of Ang II for 16 hours. The activation of AT_1_R and AT_2_R in the presence of Ang II (from 10^–9^ M to 10^–7^ M) was tested by calcium and cGMP signaling assays, respectively using the human brain microvascular endothelial cell line as an *in vitro* model.

**Results:**

Effective Ang II concentration among all tested concentrations was found to be 10^–6^ M against *Plasmodium falciparum* invasion. Statistically non-significant and negligible responses in AT_1_R and AT_2_R activation were observed below the desired Ang II concentration of 10^–6^ M.

**Discussion:**

The results indicate that the IC_50_ of Ang II against *P. falciparum* is below the threshold for AT_1_R activation. However, the minimum effective Ang II concentration required for AT_1_R activation remains unanswered which demands further improvement in experimental designs.

## Introduction

**Figure 1:**
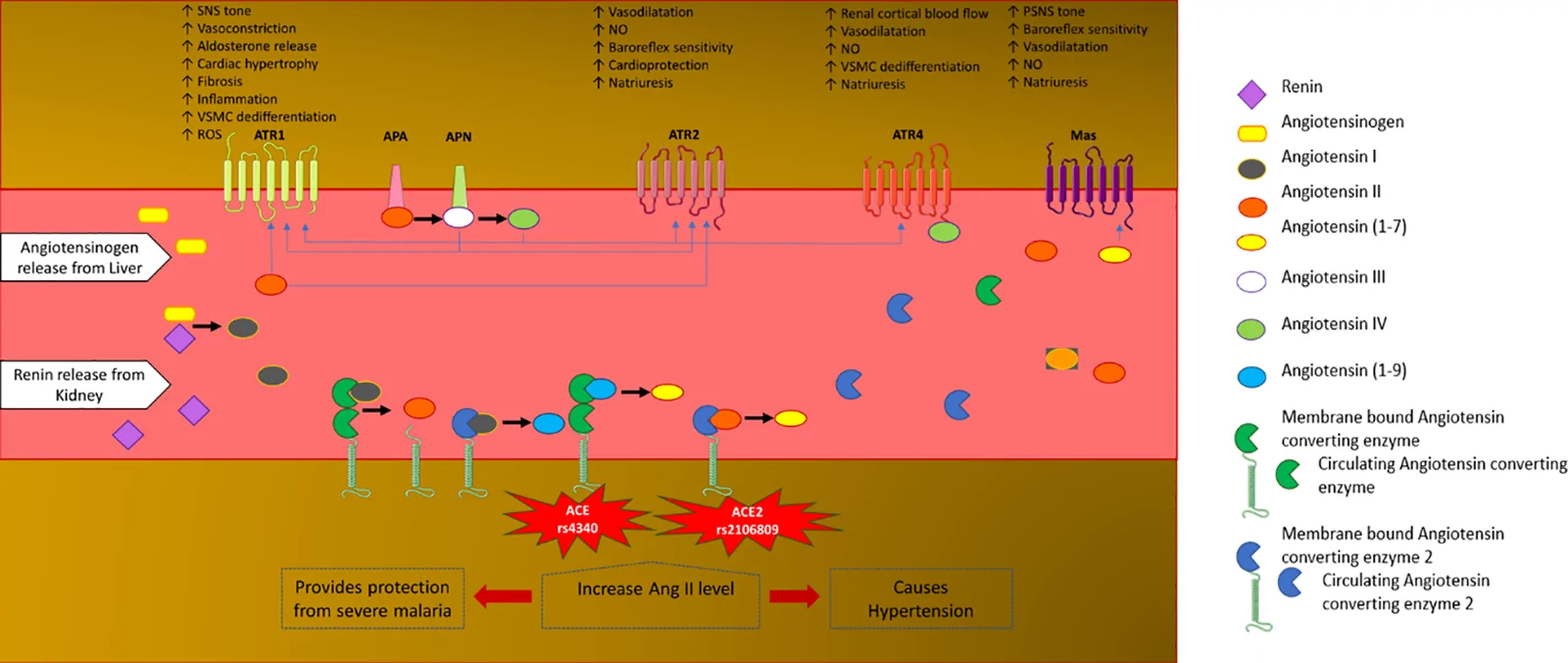
Renin-angiotensin system (RAS) with its components. Angiotensinogen (AGT) and renin (REN) are released from liver and kidney, respectively. AGT is converted to angiotensin I by REN, and angiotensin I is converted to the main regulator peptide, angiotensin II by angiotensin-converting enzyme (ACE) and also to angiotensin (1-9) via angiotensin-converting enzyme2 (ACE2). Angiotensin (1-7) is generated from angiotensin II by ACE2 and angiotensin (1-9) by ACE. Further, Aminopeptidase A (APA) cleaves angiotensin II to angiotensin III that is subsequently cleaved to angiotensin IV via Alanyl aminopeptidase N (APN). Angiotensin II acts via its 2 cognate receptors, AT_1_R and AT_2_R. Angiotensin (1-7) counterbalances AT_1_R-mediated actions through Mas receptor and also via AT_2_R.

The host-parasite interactions in the presence of chronic exposure to malaria elicit different evolutionary adaptive polymorphisms in humans (Kwiatkowski, 2005). Hypertension could be one of the detrimental health effects in presence of chronic exposure to malaria through selected polymorphisms in the Renin-Angiotensin System (RAS) (Gallego-Delgado et al., 2016b). It has been hypothesized that the evolutionarily selected polymorphisms in angiotensin-converting enzyme (ACE) and angiotensin-converting enzyme 2 (ACE2) increase Ang II levels and provide survival advantages from severe malaria while augmenting the risk of hypertension in the subsequent life span as shown in **Figure 1** (De et al., 2022; Tiwari et al., 2023).

Artemisinin-based Combination Therapies (ACTs) are the first-line treatment against malaria globally (World Health Organization, 2006). However, to achieve and sustain malaria elimination, new peptides need to be explored for their antiplasmodial activity. In this context, Ang II, a pivotal component of the human RAS that regulates blood pressure, could be one such peptide. In 2008, this compound’s anti-invasive effect was very first time studied with lower effective doses against Plasmodium (Maciel et al., 2008). Previous studies also showed that polymorphisms that lead to increased Ang II also provide direct (killing/inhibiting the growth) and indirect (via receptors) protection against severe malaria (Dhangadamajhi et al., 2010; Gallego-Delgado et al., 2015).

Ang II maintains vascular smooth muscle tone and blood volume homeostasis via its two receptors, Angiotensin II Type 1 (AT_1_R) & Angiotensin II Type 2 (AT_2_R) receptors. Ang II has also been shown to reduce erythrocyte invasion by *Plasmodium gallinaceum* (Pg; an avian malaria parasite) (Maciel et al., 2008), *Plasmodium falciparum* (Pf) (Saraiva et al., 2011) and *Plasmodium berghei* (Pb; rodent malaria parasite) (Gallego-Delgado et al., 2015). It is also known that elevated Ang II increases vasoconstriction (Li et al., 1997), hypertrophy in cardiac myocytes (Sadoshima and Izumo, 1993), inflammation (Wolf et al., 2002), neointimal vascular smooth muscle cell proliferation (Viswanathan et al., 1992), and myocardial fibrosis (Schieffer et al., 1994) by activating the AT_1_ receptor. AT_2_ receptor-mediated signaling in the presence of elevated Ang II leads to vasodilatation (Tsutsumi et al., 1999), natriuresis (Padia et al., 2008), reduced liver fibrosis (Nabeshima et al., 2006), anti-inflammatory effects (Rompe et al., 2010), etc. Thus, AT_2_ receptor-mediated responses counterbalance the AT_1_ receptor-mediated actions of Ang II (Ocaranza et al., 2006).

AT_1_ receptor is a seven-transmembrane domain protein that couples with G protein complexes (Gq/11, G12/13, Gβy) (Ushio-Fukai et al., 1999) that activate downstream signaling ultimately increasing intracellular calcium that causes smooth muscle cell contraction and high blood pressure (Ushio-Fukai et al., 1999; Yan et al., 2003; Mehta and Griendling, 2007).

G protein (Giα) coupled cyclic guanosine 3′,5′-monophosphate (cGMP), and nitric oxide (NO)-signaling cascade have been observed as functional regulation of AT_2_ receptor signaling (Yan et al., 2003; Nouet and Nahmias, 2000; Padia and Carey, 2013). It has also been reported that activation of AT_2_R in presence of AT_1_R blocker, preserves the integrity of inter-endothelial cell junctions after incubation of *P. falciparum in vitro* and protects against experimental cerebral malaria (CM) *in vivo* (Gallego-Delgado et al., 2016a). Therefore, it is suggested that Ang II may be used as an adjunct therapeutic candidate for malaria (Gallego-Delgado et al., 2016b) provided that the blood pressure homeostasis is maintained. In this context, the optimum therapeutic dose of Ang II should be more than *P. falciparum* inhibitory dose but not beyond the levels that disturbs the blood pressure homeostasis maintained through AT_1_R and AT_2_R activation. Different doses of Ang II were shown to exhibit anti-Pf activity (Padia and Carey, 2013), but the differential doses of Ang II in activating AT_1_R and AT_2_R are not well established to the best of our knowledge.

This study was hence aimed to identify the concentration of Ang II that effectively kill Pf *in vitro* and also to determine whether this concentration is greater/lesser than that needed for activation of AT_1_R and AT_2_R. The dose range of Ang II which is known to inhibit *P. falciparum* growth *in vitro* (Yan et al., 2003) has been used in this study to identify the differential activation of ATRs. As per our knowledge, this is the first *in vitro* study that is simultaneously exploring the concentration of Ang II that prevents *P. falciparum in vitro* invasion and differential activation of AT_1_R and AT_2_R in a dose-dependent manner.

## Methods

This study did not require ethics approval as the human blood used for *in vitro* culture was obtained as anonymized sample from a registered blood bank and the human brain microvascular endothelial cell line HBEC5i (ATCC^®^ CRL3245™) was commercially procured.

### *Plasmodium falciparum in vitro* culture

The cultivation of *Plasmodium falciparum* (Pf) was maintained for about 15 days as per the protocol (Trager and Jensen, 1976) before the experiment was started. Briefly, *in vitro* Pf culture was carried out in 25 cm^2^ culture flasks with a maximum of five ml of culture incubated at 37 °C in 5% carbon dioxide (CO_2_). Roswell Park Memorial Institute 1640 medium (RPMI 1640 medium) was used to culture 3D7 strain of *Plasmodium falciparum* borrowed from the Institute’s Malaria Parasite Bank. Erythrocytes infected with *Plasmodium falciparum* 3D7 strain were cultivated *in vitro* in WBC-depleted blood using RPMI 1640 medium (Gibco by Life Technologies, NY, USA) supplemented with sodium bicarbonate (2.0 g/L), glucose (2.0 g/L) 50 mg/mL Gentamicin, at 2% hematocrit with A+ human blood and human serum from AB+ blood. Giemsa-stained thin blood smears were made on microscopic glass slides every 24 hours to check parasitemia. Parasites were observed under a microscope at 100x objective lens under oil immersion (1000x magnification). The number of infected erythrocytes in a total number of 2000 RBCs after examination of at least contiguous 10 microscopic fields containing ~200 RBCs per field was noted. Percent parasitemia was calculated as per the given formula:


$$parsitemia(\%)=\frac{total\;number\;of\;infected\;RBCs}{total\;number\;of\;RBCs\;observed}\times 100$$

### Invasion inhibition assay

The maintained culture of parasites was synchronized by incubating with 5% sorbitol (w/v) in a ratio of 1:9 to obtain >98% of the parasites as ring forms (Lambros and Vanderberg, 1979). Obtained ring forms of the parasite after synchronization were cultured for ~48 hours till all parasites matured to schizonts. For the experiment, Ang II (1 mg, Sigma-Aldrich, Missouri, US) was dissolved in 1 mL nuclease-free water to get 0.9×10^–3^ M stock concentration. The prepared stock solution was used in four different final concentrations (10^–9^ M, 10^–8^ M, and 10^–6^ M and 10^–4^ M) of Ang II for the experiment. The schizonts (at 1-2% parasitemia) were incubated with these different concentrations of Ang II along with a negative control without Ang II. In addition, two positive controls chloroquine (10 μM) and medical-grade heparin (30 IU, 230 μg/mL) were also taken at the concentration they are supposed to inhibit the growth and inhibit the invasion of the parasite into RBCs. After 16 hours of incubation, with intermittent periodic examination every 3-hours, at 37 °C in 5% CO_2_, new rings, which are an indicator of recent invasion, were observed. Ring-stage parasitemia was estimated by observing 5 contiguous microscopic fields containing ~200 RBCs per field.

### Angiotensin II receptor signaling assay

Activation of AT_1_R and AT_2_R was identified by calcium and cyclic guanosine monophosphate (cGMP) signaling assay, respectively, using HBEC5i *in vitro* system (Gallego-Delgado et al., 2016a) in absence of *P. falciparum* infection. Human brain microvascular endothelial cell line HBEC5i (ATCC^®^ CRL3245™) was procured from ATCC (Manassas, VA, USA Laboratories) in dry ice that was subsequently cryopreserved in liquid nitrogen. Cell culture flask (T75 Flasks, ThermoFisher Scientific, Massachusetts, US) was coated with 7 mL of 0.1% gelatin solution (w/v) dissolved in water by incubating at 37 °C for one hour. The remaining gelatin solution was aspirated from the flask and the flask was stored at 4 °C for further use. Cryopreserved HBEC5i cells (1 mL) were thawed in a 37 °C water bath & seeded on 0.1% gelatin-coated culture flasks (T75 Flasks, ThermoFisher Scientific, Massachusetts, US). These cells were cultivated in 10ml of DMEM/F12 (Dulbecco’s Modified Eagle’s Medium and Ham’s F-12 nutrient; 1:1; v/v) medium (Cellclone-Genetix, India) at pH 7.4 and supplemented with 10% Fetal Bovine Serum (FBS; Cellclone-Genetix, India), 40 µg/ml endothelial cell growth supplement (HiMedia, India) and 1X Antibiotic-Antimycotic solution^®^ (Cellclone-Genetix, India) and incubated at 37 °C, 95% water-saturated air (5% CO_2_, 5% O_2_, 90% N_2_) in CO_2_ incubator (Cell Xpert C170™, Eppendorf™, Hamburg, Germany). Cell culture media (DMEM/F12) was replaced every three days until the cell culture achieved ~80% confluency as assessed by visual estimation under an inverted microscope (Zeiss Axiovert, Oberkochen, Germany) using a 40x objective. Viable cells were identified by trypan blue cell viability test (Strober, 2015) using 1:1 dilution of the cell suspension with a 0.4% trypan blue stain (Merck, Germany) and counted in a hemocytometer using the following formula:


$$Total\;counted\;viable\;cells\;per\;mL=\frac{total\;cells\;counted}{4\;large\;squares\;counted}\times dilution\;factor\times 10,000$$

Note:


$$\text{dilution factor}=\frac{\text{total volume}(\text{volume of cell suspension taken for cell counting}+\text{volume of trypan blue})}{\text{volume of cell suspension taken for cell counting}}$$

Cells were trypsinized in 1 mL solution (v/v) of 0.125% Trypsin-EDTA (ThermoFisher Scientific, Massachusetts, US) and sub-cultured in 1:4 ratios. All the experiments were done in a cell monolayer culture with 80% confluency, cell viability ≥90%, passage 3 and 4 (P#3 and P#4), and ~106 cells. Cell monolayer cultures were incubated for 20 min at 37 °C in a 5% CO2 incubator (Cell Xpert C170™, Eppendorf™, Hamburg, Germany) with negative control (nuclease-free water) & four concentrations (10^–9^ M, 10^–8^ M, and 10^–7^ M) of Ang II.

All experiments were performed in triplicates. Ang II-mediated activation of the AT_1_R was analyzed by calcium signaling assay (Claessens et al., 2012) using HBEC5i cell line. The supernatant was discarded from the culture of the HBEC5i cell line and 1 ml cold (4 °C) buffer (100 mM Tris, pH 7.5) was added immediately to the culture flask. Adherent cells were collected using a cell scraper and homogenized in ice (Bioruptor^®^ Plus, Diagenode, New Jersey, USA) at high power output for 10 cycles (sonication cycle: 30 secs ON, 30 secs OFF). The cell lysates were collected after centrifugation at 10,000 x g for 15 minutes at 4 °C. Intracellular Ca^2+^ present in the cell lysates was determined with a spectrophotometer using the O-Cresolphthalein Complexone calcium assay kit (Cayman Chemical, Michigan, USA). The calcium concentration was estimated as directly proportional to the intensity of purple color formed by the Calcium-Cresolphthalein Complexone Complex at 580 nm wavelength and expressed as absorbance units (A.U.) per 106 cells.

AT_2_ receptor activation in the presence of Ang II was analyzed by cGMP signaling assay (Mehta and Griendling, 2007) using HBEC5i cell line. In this experiment, the supernatant was discarded from the cell monolayer culture of the HBEC5i cell line and 2 mL 0.1 M HCl was added followed by incubation at 37 °C for 20 min in the incubator. Adherent cells were collected in microcentrifuge tubes (MCT) by scraping cells using a scraper from the culture flask. The cell suspensions were pipetted up and down to obtain a homogenous mixture of cell lysate. Homogeneous cell suspensions were centrifuged at 1000 x g for 10 minutes at 4 °C. After centrifugation, the supernatant was collected in 1.5 mL MCT for cGMP estimation using cyclic GMP ELISA Kit (Cayman Chemicals, Michigan, USA) instructions. Absorbance was measured at 415 nm wavelength. The cGMP concentration of cell lysates was calculated using competitive ELISA analysis tool (https://www.caymanchem.com/analysisTools/elisa).

Results were presented as pmol/mL of cGMP produced per 106 cells. Data were keyed into a Microsoft Excel Spreadsheet (Microsoft Office, USA) and then exported to GraphPad Prism.

### Statistical analyses

Data were expressed as mean ± SD/SEM and analyzed by one-way analysis of variance (ANOVA) in GraphPad Prism (GraphPad Prism version 8.0 for Windows, GraphPad Software, San Diego, California, USA). *Post hoc* unpaired t-test was used to make pairwise comparisons where ANOVA showed statistical significance. A p-value of <0.05 was considered statistically significant.

## Results

### Anti-invasion effects of Ang II on *P. falciparum*

**Figure 2:**
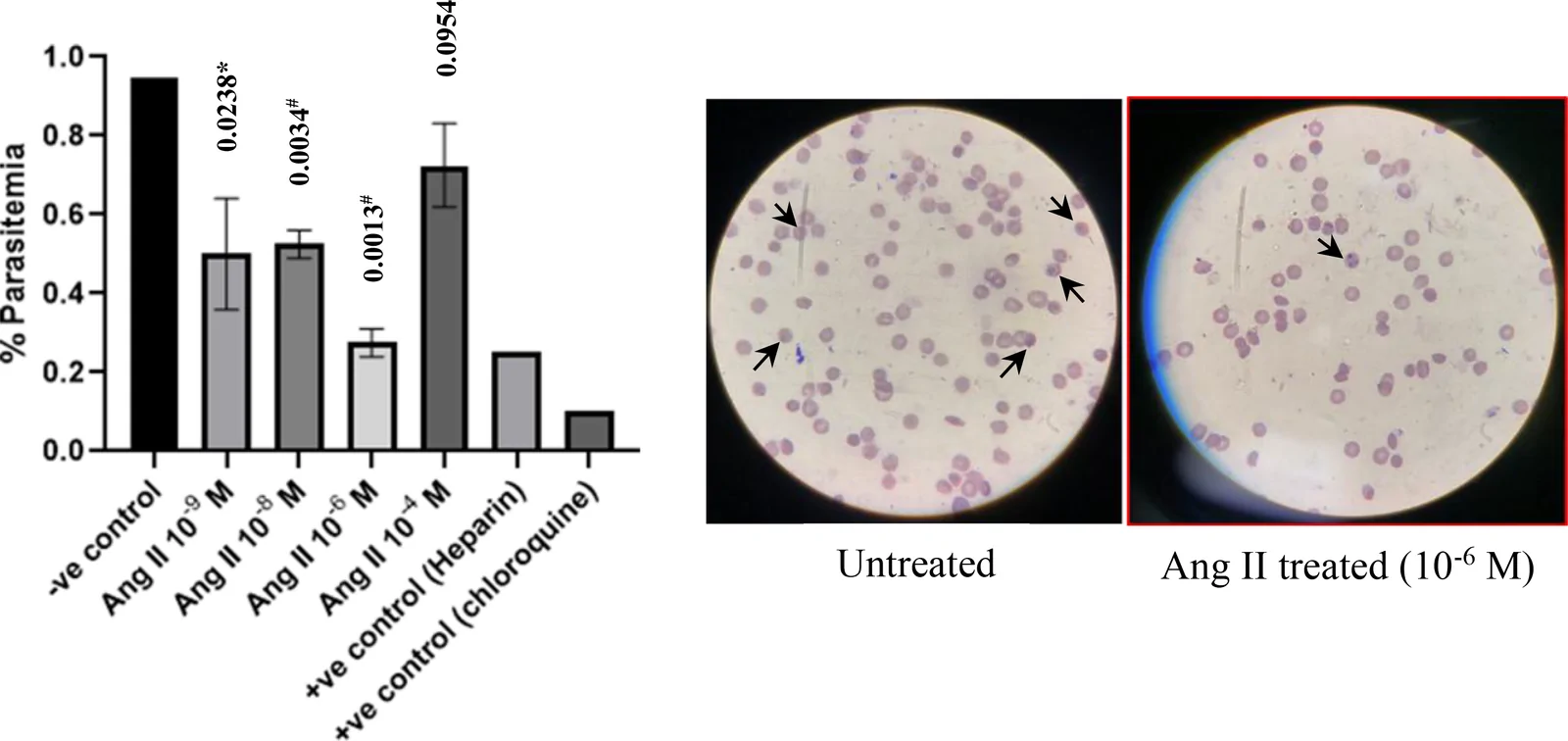
Invasion inhibition assay. Different concentration v/s percent parasitemia plotted in a bar graph chart where each bar represents mean ± standard deviation of control (negative and two positive) and 4 different concentrations (10^–9^ M, 10^–8^ M, 10^–6^ M, and 10^–4^ M) of angiotensin II. Overall statistical significance was estimated by one-way analysis of variance (ANOVA) with p-value of 0.0003 in GraphPad prism. *Post hoc* unpaired t-test revealed statistically significant difference between negative control and 10^–9^ M concentration (p<0.05; shown by *) and a highly statistically significant difference between negative control and 10^–8^ M and 10^–6^ M concentrations (p<0.01; shown by ^#^). Pairwise p-values between negative control and different Ang II concentrations are mentioned above the respective bars. A representative image of Giemsa-stained smear in untreated versus Ang II treated (10^–6^ M) is also shown on the right. The infected RBCs are shown by arrow heads.

Different concentrations of Ang II were tested against the 3D7 strain of *Plasmodium falciparum* on schizont forms for invasion inhibition. It was observed that Ang II has an anti-invasive effect on the parasite. All the tested concentrations of Ang II had a lesser percentage of ring parasitemia than the negative control. Although the results in **Figure 2**, expressed as mean ± standard deviation, show that the parasitemia was statistically significantly reduced by Ang II at 10^–9^ M (p=0.0238), 10^–8^ M (p=0.0234), Ang II at 10^–6^ M concentration was more effective against parasite invasion with the statistically significant p-value of 0.0013.

### Effects of Ang II on AT_1_ receptor activation

**Figure 3:**
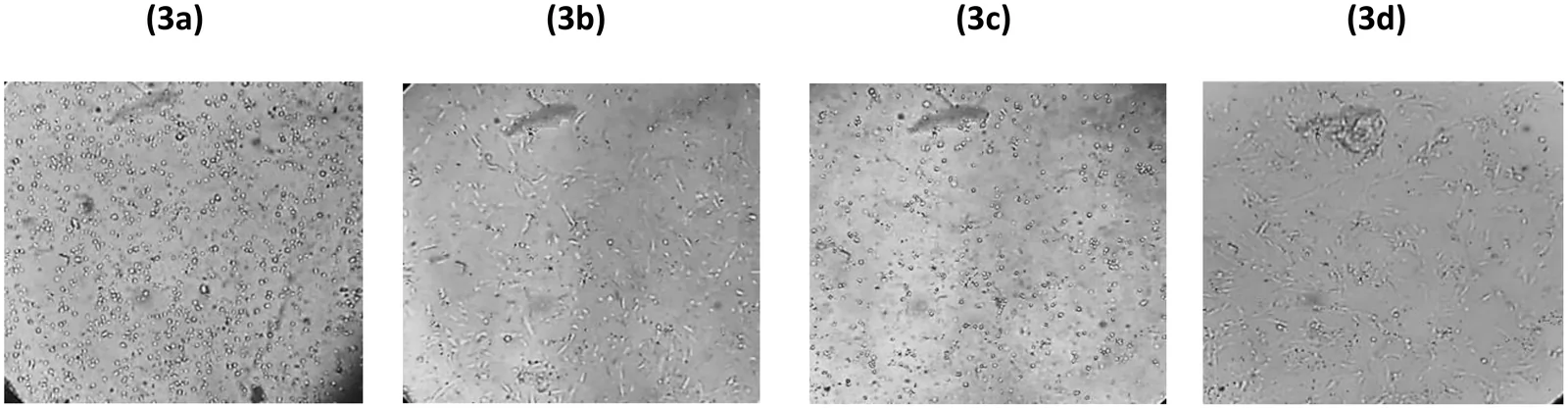
Human brain microvascular endothelial cell lines (HBEC5i) images showing the confluency of cells in hours after seeding **(a)** 7 hrs, **(b)** 24 hrs, **(c)** 36 hrs, and **(d)** 96 hrs as observed under phase contrast inverted microscopy at 400x magnification.

**Figure 4:**
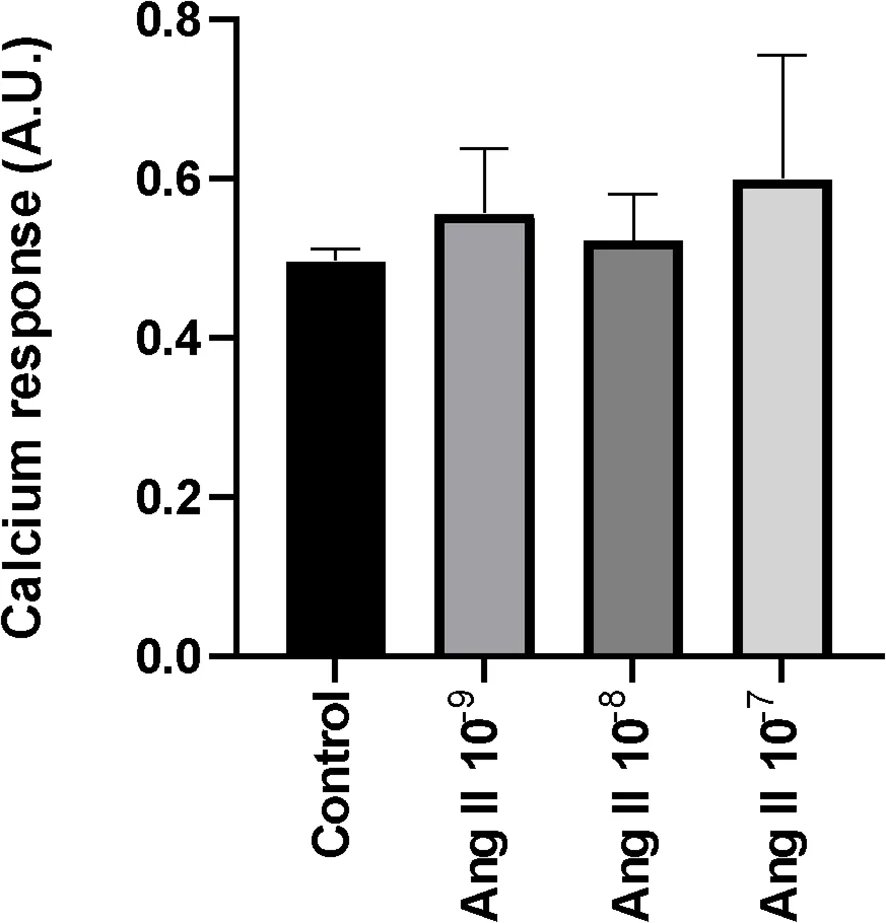
Calcium response to different doses of Ang II. The bars represent the response in the control (no angiotensin II), and at 3 different final concentrations (10^–9^ M, 10^–8^ M and 10^–7^ M) of angiotensin II. Data are expressed as mean ± SEM (Parameters: one-way analysis of variance (ANOVA) considering the p-value <0.05 as significant) in GraphPad prism.

In the calcium/cGMP assay, cryopreserved cells can be seen healthy after 6 hrs (**Figure 3a**) and the cryopreserved cells got adhered after 24 hrs. of seeding (**Figure 3b**). After 36 hrs of seeding (**Figure 3c**), most of them seeing in the floating state and all of them reached to ~80% cell adherence (confluency) at 96 hrs. after seeding (**Figure 3d**). The minimum Ang II concentrations for AT_1_ receptor activation was tested by calcium signaling assay with three different final concentrations of Ang II (10^–9^ M, 10^–8^ M, and 10^–7^ M) in the HBEC5i cell monolayer system. Statistically non-significant differences in calcium responses were observed as 0.56 A.U. 0.53 A.U. & 0.60 A.U. in three different final concentrations of Ang II (10^–9^ M, 10^–8^ M, and 10^–7^ M), respectively, as compared to that in the negative control (0.5 A.U.) (**Figure 4**).

### Effects of Ang II on AT_2_ receptor activation

**Figure 5:**
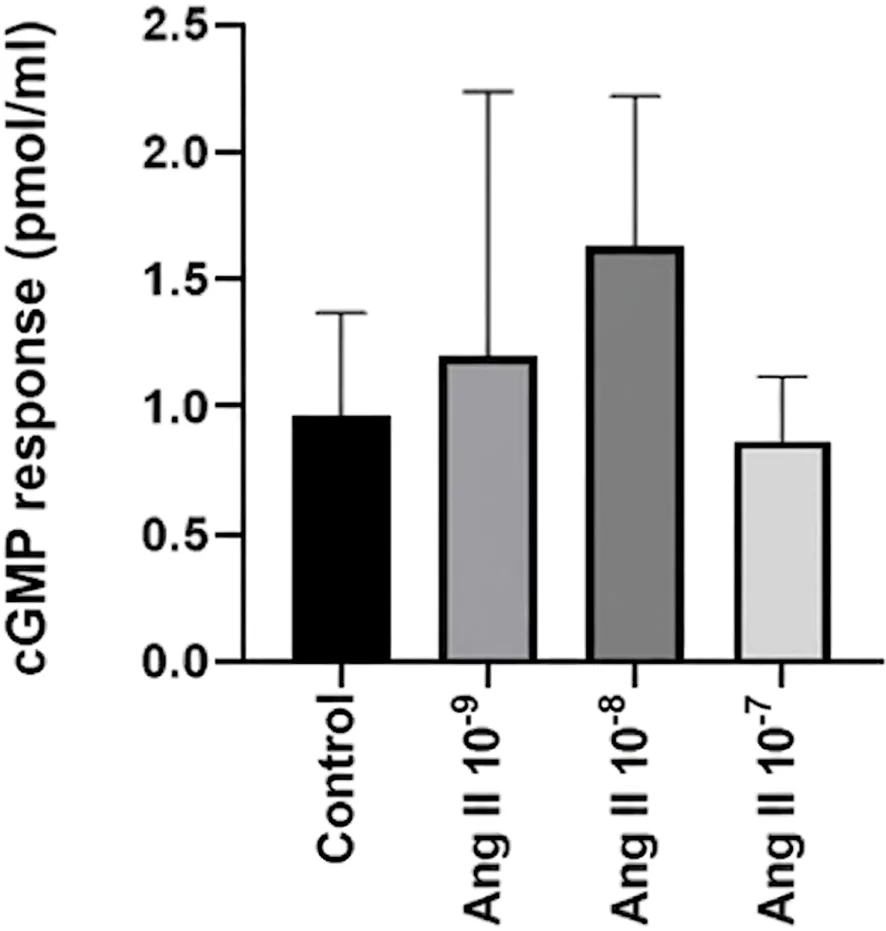
cGMP response to different doses of Ang II. The bars represent the response in the control (no angiotensin II), and at 3 different final concentrations (10^–9^ M, 10^–8^ M and 10^–7^ M) of angiotensin II. Data are expressed as mean ± SEM of different concentrations of angiotensin II. (Parameters: one-way analysis of variance (ANOVA) considering the p-value <0.05 as significant) in GraphPad prism.

The minimum dose of Ang II for cGMP response was tested by using 10^–9^ M, 10^–8^ M, and 10^–7^ M Ang II in the HBEC5i cell line. Increased responses of cGMP such as 1.19 pmol/mL and 1.66 pmol/ml were detected at 10^–9^ M and 10^–8^ M concentrations of Ang II, respectively, as compared to that in the negative control (1.00 pmol/mL; **Figure 5**), but no statistically significant difference in the response was observed.

## Discussion

The molecular and biochemical bases of the possible association between malaria and hypertension may provide some insights for evaluating adjunct therapy candidates in the form of Ang II or its analogs in malaria treatment for the population living in the malaria-endemic areas without modulating physiological blood pressure homeostasis. For this to happen, two things must be ascertained: the inhibitory concentration of Ang II for *P. falciparum in vitro* must be known and this concentration should be below the activation threshold of Ang II receptors that perturbs cardiovascular physiology. This study was conducted to find out the inhibitory concentration of Ang II and then assess the differential activation of Ang II receptors by using receptor-specific *in vitro* assay systems. In the Renin-Angiotensin System, Ang II is responsible for maintaining blood pressure in humans, and an increased concentration of Ang II is associated with hypertension. Ang II contains 8 amino acids, DRVYIHPF as its primary structure. Globally, very few and countable *in vitro* studies mostly from Brazil (while none reported from India) have been done to explore the antiplasmodial activity of Ang II (**Table 1**).

**Table 1:** Past studies to explore antiplasmodial effect of Ang II.

SN	Title (year of publication; reference)	Type of research	Country	Plasmodium species & stage	Native or analogs	Ang II concentration tested (M)	Results
1	Impairment of the *Plasmodium falciparum* erythrocytic cycle induced by Angiotensin peptides (2011; Saraiva et al., 2011)	*in vitro* (invasion inhibition)	Brazil	*Pf*	Native	10^-6^; 10^-8^; 10^-10^; 10^-12^	10^-8^ M has best inhibition
2	Angiotensin II restricted analogs with biological activity in the erythrocytic cycle of *Plasmodium falciparum* (2015; Torres et al., 2015)	*in vitro* (invasion inhibition)	Brazil	*Pf*	Ang II restricted Analogs	10^-8^	Lactam bridge restricted analogs prevented invasion
3	Effects of the angiotensin II Ala-scan analogs in erythrocytic cycle of *Plasmodium falciparum* (in vitro) and *Plasmodium gallinaceum* (ex vivo) (2015; Silva et al., 2015a)	*in vitro* (invasion inhibition) and *ex vivo*	Brazil	*Pf* and *Pg*	Native and Ala-scan Analogs	10^-8^	Only [Ala5]-Ang II presented equipotent Ang II activity
4	Angiotensin II Moderately Decreases Plasmodium Infection and Experimental Cerebral Malaria in Mice (2015; Gallego-Delgado et al., 2015)	*in vitro* (growth inhibition) and in vivo	USA	*Pf* (*in vitro*) and *Pb* (*in vivo*)	Native	10^-4^; 10^-6^; 10^-8^	10^-4^ and 10^-6^ M have better inhibition
5	New linear anti-plasmodial peptides related to angiotensin II (2015; Silva et al., 2015b)	*in vitro* (invasion inhibition)	Brazil	*Pf* & *Pg* SPZ	Native and Analogs	10^-8^	des-Asp1, Arg2, Val3, Ile5, Ang II YHPF and Ang II VIPF had better response than native Ang II
6	Angiotensin II-derived constrained peptides with anti-plasmodial activity and suppressed vasoconstriction (2017; Silva et al., 2017)	*in vitro* (invasion inhibition)	Brazil	*Pf* & *Pg* SPZ	Native and Analogs	10^-8^	Peptides CDRVYIHPFC and CDRVYHIPFC had similar response as native Ang II

The table lists 6 published studies in chronological order that explored the relation between Ang II and Plasmodium across the globe. Pf (*P. falciparum*); Pg (*P. gallinaceum*); Pb (*P. berghei*); SPZ (sporozoites).

**Figure 6:**
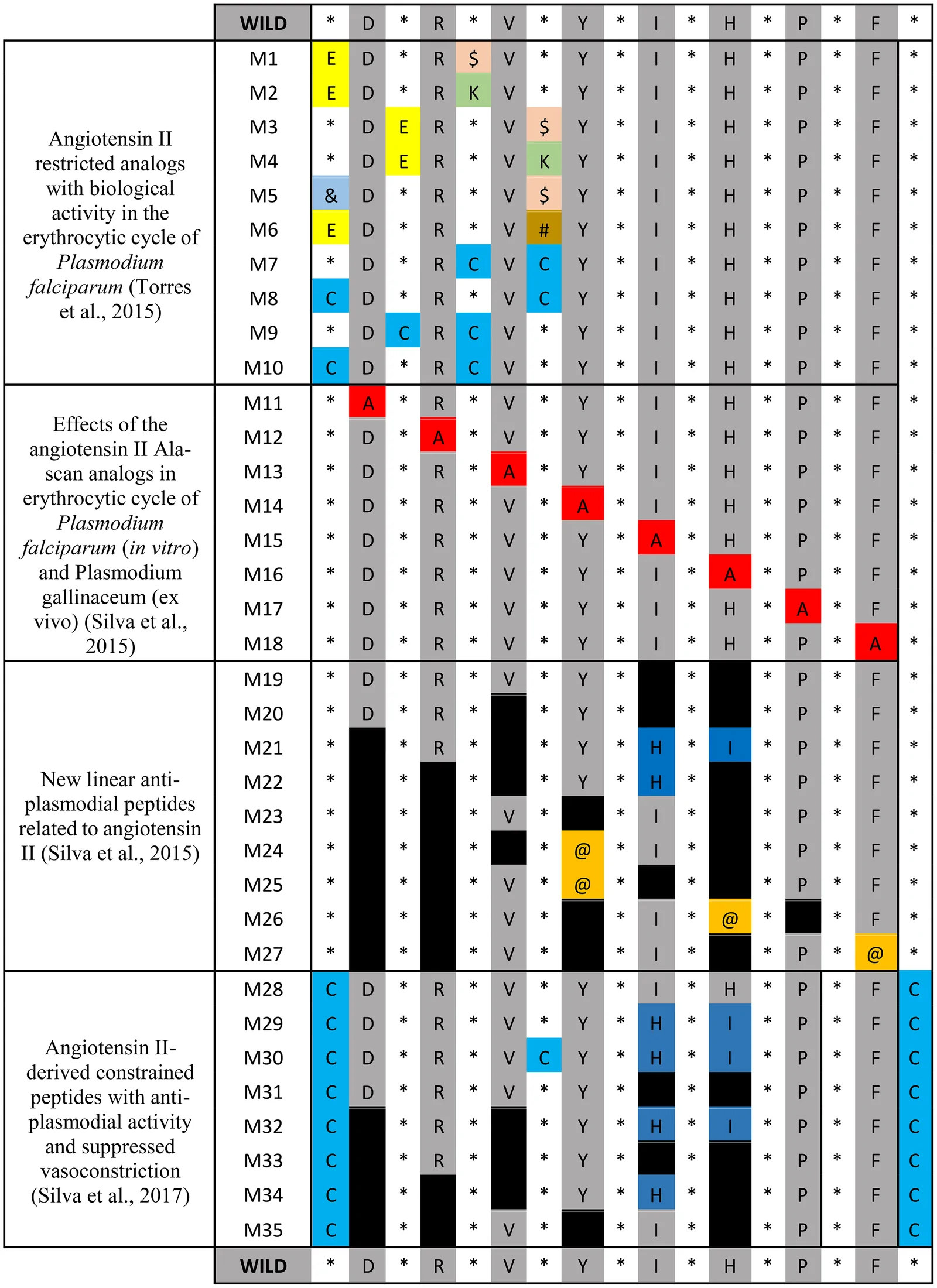
Multiple sequence alignment representation of native Ang II (wild; DRVYIHPF) and various modifications attempted in published literature. The native or unmodified octapeptide Ang II (DRVYIHPF) is depicted in the top and bottom rows of the alignment. Each of the 8 amino acid is separated by an asterisk to accommodate any downstream amino acid modification. The asterisk is also place before and after the first and last amino acid as well for the same. Amino Acid modifications are shown as coloured boxes with alphabets (amino acid substitutions), and special characters (for any other specified modification). Deleted amino acid loci are shown as black boxes. A (alanine), R (arginine), D (aspartic acid), C (cysteine), E (glutamic acid), G (glycine), H (histidine), I (isoleucine), K (lysine), F (phenylalanine), P (proline), Y (tyrosine), V (valine), $ (ornithine), # (D-ornithine), & (D-glutamic acid), @ (2,4-Di¬nitro-N-(2-phenyl¬ethyl) aniline). M1-M35 indicate the different type of modifications.

Its effect at various concentrations was previously checked for the very first time on *Plasmodium gallinaceum* in 2008 (Maciel et al., 2008) and *Plasmodium falciparum* in 2011 (Saraiva et al., 2011). Various analogs/synthetic peptides of Ang II have also been studied to check their activity against Pf and Pg (**Figure 6**, **Table 1**).

A bunch of work has been done to change its primary structure to make it more feasible to use as an anti-malarial compound but only a couple of studies have been done for identifying its effective concentration (**Table 1**). This component has also been suggested as an adjunct treatment for severe malaria (Gallego-Delgado et al., 2016b). In the current study, the range of 10^–6^ to 10^–8^ M Ang II seems to be more effective as an anti-invasive agent. The anti-parasitic effects of Ang II are more suggestive of an invasion defect rather than killing of the ring stage parasites as tightly synchronized late-stage parasites (matured schizonts) were used as the starting parasite culture and the cultures were monitored every three hours for 16-hours. We believe that the reduced ring stage parasitemia was more likely to be due to reduced invasion as no dead ring stage parasites could be observed during the entire monitoring. While we cannot completely exclude minor effects on early ring-stage development, the primary readout corresponds more likely to an invasion defect.

Further, we observed a significant reduction in parasitemia even at lower concentrations (10^-9^ M), with maximal inhibition at 10^-6^ M. Interestingly, at the highest concentration tested (10^-4^ M), the inhibitory effect was not sustained, and it seems that an increase in parasitemia was noted. This phenomenon is consistent with previously reported findings where Saraiva et al. (2011) demonstrated that the angiotensin peptides modulate the *P. falciparum* erythrocytic cycle in a complex and non-linear manner, likely mediated through receptor-dependent and receptor-independent pathways. At higher concentrations, Ang II may exhibit altered biological effects, including receptor desensitization, activation of alternative signaling pathways, or loss of specificity, which can attenuate the inhibitory effect observed at optimal concentrations.

Additionally, it is important to note that the variability at 10^-4^ M in our experiments was relatively high, as reflected by an increased standard deviation. This suggests reduced reproducibility at supra-physiological concentrations, possibly due to non-specific effects, peptide aggregation, or experimental variability. Taken together, these observations indicate that the maximal inhibitory effect occurs within an optimal concentration range (around 10^-6^ M), while higher concentrations may not proportionally enhance efficacy and may instead introduce variability or off-target effects.

In addition, the difference in inhibitory concentrations between our study (10^-6^ M) and the findings reported by prior studies (Saraiva et al., 2011) (10^-8^ M) warrants further clarification. This discrepancy may be attributed to several methodological and biological factors. First, differences in experimental conditions such as cell type/parasite strain used, assay system, incubation time, and endpoint measurement—can significantly influence the observed inhibitory concentrations. In our study, we used 3D7 strain (W2 strain in previous studies) revived using sorbitol method, which may require higher concentrations to achieve comparable inhibitory effects.

Second, variations in compound preparation, stability, incubation period and bioavailability under our experimental conditions could also contribute to the observed difference in effective concentration. Additionally, differences in readout sensitivity between assays (e.g., viability-based such as microscopy vs. molecular endpoints) may further account for this variation.

Another important point to consider here is that the presence of ACE2 in FBS or blood may catalyze the conversion of Ang II to Ang 1–7 and other downstream metabolites. This enzymatic activity has been previously reported, including by Saraiva et al. (2011), who demonstrated that Ang II can be rapidly metabolized in biological systems, thereby influencing the observed biological responses and Ang-(1–7) has the same but non-additive effect as Ang II.

In the context of our invasion inhibition assays, where human blood and serum was used as a supplement, it is indeed plausible that partial degradation of Ang II may have occurred. Consequently, we acknowledge the possibility that the observed anti-invasive effects may not be exclusively mediated by Ang II itself, but could also involve its bioactive metabolites, such as Ang 1–7 or Ang 1–5. These peptides are known to exert biological effects distinct from, and in some cases opposing, those of Ang II.

Furthermore, the use of serum-containing conditions was intentional to support optimal parasite growth and maintain physiological relevance. Serum-free media are known to impair parasite viability and invasion efficiency, which could confound the interpretation of results by introducing growth-related artifacts rather than reflecting true invasion inhibition (Willet and Canfield, 1984; Basco, 2023). Thus, the absence of serum may itself act as a confounding factor, making it difficult to distinguish whether reduced invasion is due to treatment effects or compromised parasite fitness.

After demonstrating the results with the approach to explore the different concentrations of Ang II against RBC invasion of Plasmodium, these concentrations were used to activate angiotensin receptors. The receptor-activation concentration of Ang II was achieved by analyzing the preferential activity of Ang II on its two cognate receptors AT_1_R and AT_2_R by GPCR PLC-mediated calcium signaling pathway and GPCR cGMP signaling pathway, respectively, using an *in vitro* model (human brain microvascular endothelial cell line; HBEC-5i; ATCC^®^ CRL-3245™) where both receptors are present (Gallego-Delgado et al., 2016a). The HBEC5i is an immortalized cerebral microvascular endothelium cell system isolated from the human cerebral cortex. This cell line is suitable for analyzing microvascular changes of the brain caused by malaria (Claessens et al., 2012).

Ang II triggers GPCR Phospholipase C mediated signaling pathway that leads to phosphatidylinositol hydrolysis, the formation of inositol triphosphate (IP3), and diacylglycerol accumulation (DAG) through the AT_1_ receptor. IP3 evokes a subsequent increase in intracellular calcium. Ang II induces intracellular cyclic guanosine monophosphate (cGMP) release which induces nitric oxide (NO)-signaling pathway through the AT_2_ receptor. Based on previously tested Ang II concentration ranges, 10^–9^ to 10^–6^ M Ang II (Gebke et al., 1998; Caputo et al., 1995; Suarez et al., 2002; Hannan et al., 2004) for AT_1_ and AT_2_ receptor signaling using various cell systems and 10^–8^ M and 10^–6^ M for antiplasmodial activity (Saraiva et al., 2011), three different final concentrations of Ang II were selected for this study. Receptor activation was observed at 10^–9^ M Ang II dose which is below the Pf inhibitory dose. However, the result is inconclusive due to lack of statistically significant difference with the negative control. A statistically significant difference of receptor responses with negative control using a wider range of Ang II concentrations may identify whether the receptor activation dose is below or above the Pf inhibitory dose.

Although the calcium responses were observed in three different concentrations of Ang II (10^–9^ M, 10^–8^ M, and 10^–7^ M), statistically non-significant differences with the negative control made the result inconclusive for the minimum dose of Ang II to activate AT_1_ receptor. Previous studies on human brain endothelial cells (Claessens et al., 2012) and neurons (Suarez et al., 2002) showed calcium response at 10^–7^ M and 10^–9^ M Ang II, respectively. Both the studies were done using fluorescence-based calcium assays which are high throughput assays as compared to the colorimetric-based calcium detection assay used in this study. The cGMP responses were observed in 10^–9^ M and 10^–8^ M of Ang II but a statistically non-significant difference was observed in comparison to control. As per the literature survey, cGMP responses were also observed at 10^–6^ M and 10^–9^ M Ang II in rat carotid arteries (Caputo et al., 1995) and urinary arteries (Hannan et al., 2004), respectively. It is reported that ACE2 present in FBS degrades Ang II to ang (1-7) (Lubel et al., 2008), and therefore decreasing Ang II may be unable to stimulate either calcium or cGMP signaling cascade. It has also been studied that heterodimer formation of Ang II receptors with other receptors and/or between themselves may inhibit receptor activity (Rukavina et al., 2020).

## Conclusions

Out of the concentrations of Ang II tested (10^–4^ to 10^–9^ M) for inhibition of *P. falciparum* 3D7 invasion into RBCs, Ang II at 10^–6^ M concentration was significantly more effective. No statistically significant activation of AT_1_R and AT_2_R could be observed at 10^–9^ M, 10^–8^ M, and 10^–7^ M Ang II concentrations, that is, below the desired Ang II concentration of 10^–6^ M, suggesting that the IC_50_ of Ang II against *P. falciparum* is below the threshold for AT_1_R activation. However, the minimum effective Ang II concentration required for AT_1_R activation remains unanswered.

## Limitations and way ahead

There are a few limitations of this study: no positive control for Ang II activation was used, AT_1_ and AT_2_ receptor blockers were not used, and experiments were performed in media supplemented with fetal bovine serum (FBS). Further, experiments can be designed to activate the AT_2_R in the presence of an AT_1_R blocker (and vice-versa) without the chances of heterodimer-mediated receptor masking. A higher concentration of Ang II may be required that can maximally stimulate AT_1_R and AT_2_R and act as a positive control for Ang II-mediated receptor activation. Moreover, studies with FBS-free media, wider Ang II concentration ranges (10^–11^ M to 10^–4^ M), and higher throughput detection assays may rule out the chances of confounding factors and provide a consistent conclusive result. It is also suggested that more biological replicates and using a more robust assay platform may be needed in future studies to detect minute dose responses.

## Data Availability

The original contributions presented in the study are included in the article/supplementary material. Further inquiries can be directed to the corresponding author.
